# The Reversal of Memory Deficits in an Alzheimer’s Disease Model Using Physical and Cognitive Exercise

**DOI:** 10.3389/fnbeh.2020.00152

**Published:** 2020-08-21

**Authors:** Leticia R. Dare, Alexandre Garcia, Caroline B. Soares, Luiza Lopes, Ben-Hur S. Neves, Daniel V. Dias, Pâmela B. Mello-Carpes

**Affiliations:** ^1^Physiology Research Group, Federal University of Pampa, Uruguaiana, Brazil; ^2^Department of Structural Biology, Federal University of Triangulo Mineiro, Uberaba, Brazil

**Keywords:** Alzheimer’s disease, physical exercise, cognitive exercise, oxidative damage, Aβ neurotoxicity

## Abstract

Alzheimer’s disease (AD) is the leading cause of dementia in the world, accounting for 50–75% of cases. Currently, there is limited treatment for AD. The current pharmacological therapy minimizes symptom progression but does not reverse brain damage. Studies focused on nonpharmacological treatment for AD have been developed to act on brain plasticity and minimize the neurotoxicity caused by the amyloid-beta (Aβ) peptide. Using a neurotoxicity model induced by Aβ in rats, the present study shows that physical (PE) and cognitive exercise (CE) reverse recognition memory deficits (with a prominent effect of long-term object recognition memory), decrease hippocampal lipid peroxidation, restore the acetylcholinesterase activity altered by Aβ neurotoxicity, and seems to reverse, at least partially, hippocampal tissue disorganization.

## Introduction

According to the World Health Organization, Alzheimer’s disease (AD) is a global public health priority (Lane et al., 2018). This neurodegenerative disease is the most common form of dementia, accounting for 50–75% of cases (Prince et al., 2015). AD is related to aging, and every 5 years after 65 years of age, its prevalence doubles (Prince et al., 2015).

The formation and aggregation of abnormal amyloid-beta (Aβ) peptides in the extracellular space, hyperphosphorylated tau protein, and brain oxidative stress are some of the pathological alterations found in AD (Grundke-Iqbal et al., 1986; Selkoe, 1999; Lee et al., 2001; Moneim, 2015). These alterations lead to a gradual loss of cognitive function, usually starting with short-term memory (STM) dysfunction, impaired judgment and reasoning, and disorientation and culminating in total memory loss and personality alterations (Martins et al., 2018).

In addition to the high prevalence of AD, there are limited options for treatment for this disease. Furthermore, the current pharmacological therapies only minimize the progression of the symptoms; they do not reverse brain damage (Habtemariam, 2019). In this sense, research focused on nonpharmacological treatment has been developed (Zucchella et al., 2018). Among the possible nonpharmacological strategies is physical exercise (PE).

PE improves cerebral blood circulation, thereby increasing the supply of oxygen and energetic substrates to the brain (Black et al., 1991). The effects of aerobic exercises, such as running on a treadmill, can also be related to its effect on reducing the formation of amyloid plaques and the hyperphosphorylation of tau and on reducing neuroinflammation and oxidative stress (Dao et al., 2014; Koo et al., 2017; Lu et al., 2017).

Another potential nonpharmacological treatment is cognitive exercise (CE). Cognitive training contributes to maintaining neural functions, promoting cognitive flexibility, decreasing oxidative stress, and improving the quality of life in patients. Evidence suggests that CE produces long-lasting improvements in the memory performance of older adults who experience a normal cognitive decline (Winocur et al., 2007). Previous studies have demonstrated that cognitive stimulation programs are effective in maintaining cognition and quality of life in AD patients with mild to moderate dementia (Woods et al., 2012; Epperly et al., 2017). Despite these positive observations, the mechanisms by which CE acts in the AD brain are not yet well described. Recently, using an animal model of Aβ neurotoxicity, we showed that CE is effective in protecting against memory deficits when it is performed before neurotoxicity induction (Rossi Dare et al., 2019). In this case, we observed that CE was able to avoid the brain oxidative imbalance induced by Aβ.

One of the main properties of the brain is neuroplasticity. The term “neuroplasticity” refers to the ability of the organ to change according to external stimuli, altering its function and morphology through neural mechanisms such as synaptogenesis and neurogenesis (Lövdén et al., 2013; Calabrese et al., 2014). Learning and memory are examples of functions that are highly dependent on hippocampal neuroplasticity; deficits in this process, as occur in AD, lead to memory impairments (Yau et al., 2015). Cognitive plasticity may be strengthened *via* aerobic (Foster et al., 2011) and cognitive training (Greenwood and Parasuraman, 2010).

It is important to highlight that our group has already demonstrated that CE is as good as PE as a preventive strategy for memory deficits related to Aβ neurotoxicity (Rossi Dare et al., 2019). Despite the well-demonstrated preventive effects of PE and CE, we investigated whether the same strategies could be used to treat memory deficits. In the present case, PE and CE training were introduced after the induction of Aβ neurotoxicity, when cognitive deficits were already established. Our results demonstrate that PE and CE can reverse memory deficits, hippocampal oxidative imbalance, and some hippocampal morphological alterations related to Aβ neurotoxicity.

## Materials and Methods

### Animals and Experimental Design

All experiments were carried out according to the Principles of Laboratory Animal Care and in agreement with the guidelines established by the Local Institutional Animal Care and Use Committee (approved protocol n. 14/2017). Adult male Wistar rats were purchased from the Federal University of Santa Maria (RS/Brazil) and were housed at the institute’s vivarium under controlled temperature (23 ± 2°C) in a 12-h light-dark cycle, with food and water available *ad libitum*.

**Figure 1:**
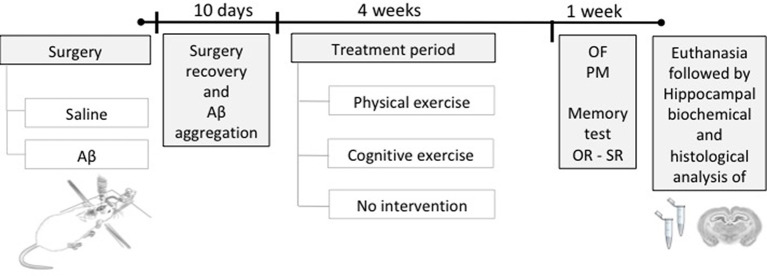
Experimental design. Rats were submitted to a stereotaxic surgery with the injection of Aβ or saline solution (sham surgery) in the hippocampus. Ten days after surgery, the time required for surgery recovery and Aβ aggregation, the training period started, being: physical exercise (PE), cognitive exercise (CE), or none (no intervention), during 4 weeks. Afterward, behavioral tests were performed to evaluate the object and social recognition memory and to monitor the general behavioral; after, the euthanasia was performed to brain tissue collection for histological and biochemical analyses. OR, object recognition memory test; SR, social recognition memory test; OF, open field; PM, plus maze.

Initially, the animals were divided into two large groups and were subjected to stereotaxic surgery for intrahippocampal infusion of Aβ protein or saline (vehicle), followed by 10 days of recovery from surgery and Aβ aggregation. After this period, animals were subdivided according to the treatments (PE training, CE training, or no treatment), resulting in six groups (*n* = 12/group): sham surgery (Control); Aβ-induced neurotoxicity model (Aβ); sham surgery and PE training (PE); sham surgery and CE training (CE); Aβ and PE training (Aβ + PE); and Aβ and CE training (Aβ + CE). After that, the animals were subjected to behavioral tests to evaluate recognition memory and to monitor control parameters. Finally, the rats were euthanized, and brain tissue was collected for biochemical testing (*n* = 8) or histological analyses (*n* = 4; Figure 1).

### Aβ-Induced Neurotoxicity Model

Aβ peptide 25–35 (A4559; Sigma–Aldrich) was dissolved in saline solution (i.e., vehicle) at a concentration of 100 μM and incubated at 37°C for 4 days to induce Aβ 25–35 aggregation initiation. A total volume of 2.0 μl of Aβ protein was injected into each hippocampus based on the Paxinos and Watson brain atlas coordinates (anterior-posterior = −4.2 mm; lateral–lateral, ± 3.0 mm; ventral-medial, −3.0 mm) by stereotaxic microinjection using a Hamilton syringe and an infusion pump. After surgery, rats were returned to their home cages and were monitored for 10 days.

### Treatments

#### Physical Exercise (PE)

Before PE training, the rats were habituated to a treadmill built for rodents (Insight Limited, São Paulo, Brazil) to avoid stress effects. The habituation was conducted for 3 days (on the first day, the animals were placed on the treadmill turned off for 10 min; on the second and third days, they were put in the treadmill at a velocity of 2–5 m/min for 10 min).

After the rats were subjected to the “good runner protocol,” which consists of placing the animals on the treadmill without inclination for three consecutive days (velocity 8 m/min for 10 min), the level of trainability was evaluated with a score ranging from 1 to 5 points (1: refuses to run; 2: below the average of the runners—runs and stops or runs in the wrong direction; 3: average runner; 4: above the average—runs well, with sporadic stops; 5: good runner—runs and always stays in the front part of the treadmill). In the end, the animals that maintained an average of three or more points were included in the exercise group.

On the last day, an indirect oxygen consumption (VO_2_) running test was performed to determine the individual intensity of exercise. For this, the rats started running at a low velocity, which was increased 5 m/min every 3 min until the rat was unable to keep running. The time to fatigue (min) and the work volume (m/min) were considered indirect measures of maximal indirect VO_2_ (Brooks and White, 1978).

The PE started in the following week and was performed at an intensity of 60–70% of the maximal indirect VO_2_ (Cechetti et al., 2012; Malek et al., 2013), three times a week, once a day for 30 min, for 4 weeks, without treadmill inclination.

#### Cognitive Exercise (CE)

The CE was based on the adaptation of the Barnes maze memory task (Barnes, 1979) as proposed by Rossi Dare et al. (2019). The Barnes maze is a circular platform with 20 potential escape holes, equally spaced in the periphery, only one of which leads to an escape cage. Negative reinforcement (bright lights) is used to motivate the animal to escape to a dark cage hidden underneath one of the holes. Visual cues surrounding the maze are used to make spatial learning possible.

To perform the CE, the animals were trained every day in the Barnes maze for 4 weeks, and each day, they were able to perform the CE more efficiently, i.e., they found the escape hole more quickly using the spatial cues. Therefore, every 10 days, the escape cage was altered to another place; consequently, the animals had to form a new spatial memory, which required cognitive flexibility (Rossi Dare et al., 2019).

### Control Behavioral Tasks

The open field (OF) and elevated plus maze (PM) were used to analyze exploratory and locomotor activities and to evaluate anxiety state, respectively.

In the OF, the rats were placed in the left quadrant of a 50 × 50 × 39 cm open field made with wood painted white, with a frontal glass wall. Black lines were drawn on the floor to divide it into 12 equal quadrants. Crossing and rearing, as measures of locomotor and exploratory activities, respectively, were measured over 5 min (Bonini et al., 2006).

In the PM, the rats were placed in the center of the maze. The maze consists of two open arms (50 × 10 cm) and two enclosed arms (50 × 10 × 40 cm), with an open roof, arranged such that the two open arms were opposite to each other. The maze is elevated to a height of 50 cm. The total number of entries and the time spent in the four arms were recorded over a 5-min session (Pellow et al., 1985).

### Memory Tests

#### Object Recognition (OR)

Rats were first habituated individually to the OR memory task apparatus and left to freely explore it for 20 min during four consecutive days before the training session. On the fifth day, OR memory training was performed. In the training, two different novel objects were placed in the apparatus, and rats were allowed to freely explore them for 5 min. Three hours and 24 h later, STM and long-term memory (LTM) were evaluated, respectively (Broadbent et al., 2010). In each testing session, one of the objects was randomly replaced by a novel/unfamiliar object, and the rats were reintroduced into the apparatus for an additional 5 min period of free exploration. The time spent exploring the familiar and novel objects was recorded. Additionally, the discrimination index (DI) on STM and LTM tests was determined by the difference of time spent exploring the new (T novel) and the familiar (T familiar) objects: DI = [(T novel − T familiar)/(T novel + T familiar) × 100 (%)], and used as a memory parameter.

#### Social Recognition (SR)

The SR memory task is an adaptation of the social interaction test proposed by Kaidanovich-Beilin et al. (2011). The task was completed in 3 days. First, the rats were placed in an arena with two small cages for 20 min for habituation to the apparatus. On the following day, a training session was performed with the inclusion of one unfamiliar rat in one of the cages for 10 min of free exploration. After 24 h, a testing session was performed when the same rat from the training (now a familiar rat) and a new/unfamiliar rat was placed for exploration for 10 min. The time spent exploring the familiar and novel rats was recorded. Exploration of the conspecific animal was defined as sniffing or touching the small cages with the nose and/or forepaws. Additionally, the DI was determined by the difference of time spent exploring the unfamiliar (T novel) and the familiar (T familiar) rat: DI = [(T unfamiliar − T familiar)/(T unfamiliar + T familiar) × 100 (%)], and used as a memory parameter.

### Biochemical Testing

After euthanasia, the brain tissues of some animals (*n* = 8) were quickly removed, and then the hippocampal tissues were immediately isolated from the brain and cleaned using ice-cold saline. Tissue samples were frozen in liquid nitrogen and stored at −80°C until biochemical analysis was performed.

For biochemical experiments, the tissue samples were homogenized in 50 mM Tris-HCl, pH 7.4. The homogenates were centrifuged at 2,400 *g* for 20 min at 4°C to obtain supernatants that were used for the analysis of all biochemical variables.

Hippocampal reactive oxygen species (ROS) levels were measured by a spectrofluorometric method using 20,70-dichlorofluorescein diacetate (DCFH-DA; Loetchutinat et al., 2005). The sample was incubated in darkness with 5 μl of DCFH-DA (1 mM). The oxidation of DCHF-DA to fluorescent dichlorofluorescein (DCF) was measured for the detection of intracellular ROS. The formation of the oxidized fluorescent derivative (i.e., DCF), measured by DCF fluorescence intensity, was recorded at 520 nm (480-nm excitation) 30 min after the addition of DCFH-DA to the medium. The results are expressed as arbitrary units.

The hippocampal lipid peroxidation level was evaluated by the TBARS test (Ohkawa et al., 1979). The samples were incubated with a 0.8% thiobarbituric acid solution, acetic acid buffer (pH 3.2), and SDS solution (8%) at 95°C for 2 h and the color reaction was measured at 532 nm. The results were expressed as nanomoles of malondialdehyde per milligram of protein.

The total antioxidant capacity was measured by FRAP (ferric reducing/antioxidant power) assay. The working FRAP reagent was prepared by mixing 25 ml acetate buffer, 2.5 ml TPTZ solution, and 2.5 ml FeCl_3_·6H_2_O solution. The homogenate (10 μl) was added to 300 μl of working FRAP reagent in a microplate (Benzie and Strain, 1996). Additionally, a standard curve with 10 μl Trolox concentrations (15, 30, 60, 120, and 240 mM) and 300 μl working FRAP reagent was used. The microplate was incubated at 37°C for 15 min before reading in a SpectraMax M5 Microplate Reader at 593 nm.

Acetylcholinesterase (AChE) activity is a marker of the loss of cholinergic neurons in the forebrain. The AChE activity was assessed by the Ellman method (Ellman et al., 1961). The reaction mixture was composed of 100 mM phosphate buffer, pH 7.4, and 1 mM 5,5′-dithio-bis-2-nitrobenzoic acid. The method is based on the formation of a yellow anion, 4,4′-dithio-bis nitrobenzoic acid, after the addition of 0.8 mM acetylthiocholine iodide. The change in absorbance was measured for 2 min at 30-s intervals at 412 nm (SpectraMax M5; Molecular Devices). The results were expressed as micromoles of acetylthiocholine iodide hydrolyzed per minute per milligram of protein.

### Histological Analysis

Some rats (*n* = 4) were anesthetized and transcardially perfused with phosphate-buffered saline (PBS) solution followed by 4% formaldehyde. Brains were removed, postfixed for 24 h in 4% formaldehyde, and cryopreserved in 30% sucrose overnight at 4°C. Then, the brains were frozen, and coronal brain sections (12 μm thickness) were cut in a Cryostat (LEICA CM3050S). The sections were stained by hematoxylin-eosin (HE), and a qualitative analysis of the morphological parameters were observed under an optical microscope (Olympus CX21).

### Statistical Analysis

First, the data normality was evaluated by the Shapiro–Wilk test. Behavioral results are expressed as the mean ± the SD. Object exploration time in the OR memory task and rat exploration time in the SR memory task were converted to a percentage of total exploration time, and a one-sample *t*-test was used to compare the percentage of the total time of exploration spent on each object/rat with a theoretical mean of 50%. The OR STM DI and the SR DI data were compared between the groups using one-way ANOVA. The OR LTM DI data were compared between the groups using one-way Kruskal–Wallis followed by the Mann–Whitney test. The OF and PM data were analyzed by one-way ANOVA.

Biochemical results that followed a normal distribution (DCFH and TBARS) were compared using ANOVA followed by Tukey’s *post hoc* test and are expressed as the mean ± the SD. For non-normal variables, AChE and FRAP, a Kruskal–Wallis test was performed, followed by Dunn’s *post hoc* test; these data are expressed as the median ± the interquartile range.

The significance level was set at 0.05 for all variables.

## Results

### Control Behavioral Tasks

There were no differences between the groups in the number of rearings (*F*_(5,64)_ = 1.171; *P* = 0.33; Table 1) and crossings (*F*_(5,64)_ = 3.279; *P* = 0.36; Table 1) during the free exploration session in the OF, showing that treatments and surgery did not affect rats’ exploratory and locomotor behavior. In the same way, the procedures did not affect anxiety behavior, since no differences were observed among groups in the PM test (*F*_(5,64)_ = 0.7539; *P* = 0.58). These data are important since they guarantee that the results observed on memory tasks are related to procedures influencing learning and memory processes and not to other behavioral alterations.

**Table 1:** Different training and surgery procedures do not alter the locomotor and exploratory activities evaluated in the open field, and the anxiety behavior evaluated in the elevated plus maze.

	Control	Aβ	PE	Aβ + PE	CE	Aβ + CE	*P*-value
**Open field**					
Rearings (*n*)	33.4 ± 9.58	33.2 ± 6.85	39.4 ± 12.13	40.4 ± 10.24	37.6 ± 7.39	39.0 ± 11.30	0.33
Crossings (*n*)	98.5 ± 27.73	108.0 ± 16.43	98.9 ± 18.13	124.2 ± 24.10	125.3 ± 20.36	118.0 ± 17.25	0.09
**Plus maze**					
Time in open arms (s)	194.9 ± 45.37	221.1 ± 41.07	200.6 ± 32.36	197.5 ± 48.16	213.8 ± 37.74	211.7 ± 39.56	0.58

*Data are expressed as mean ± SD of the number of crossings and rearings on the OF, and of the percentage of time spent in the open arms of the PM (*n* = 12 per group; *P* > 0.05; one-way ANOVA)*.

### Memory Tasks

#### Object Recognition (OR)

**Figure 2:**
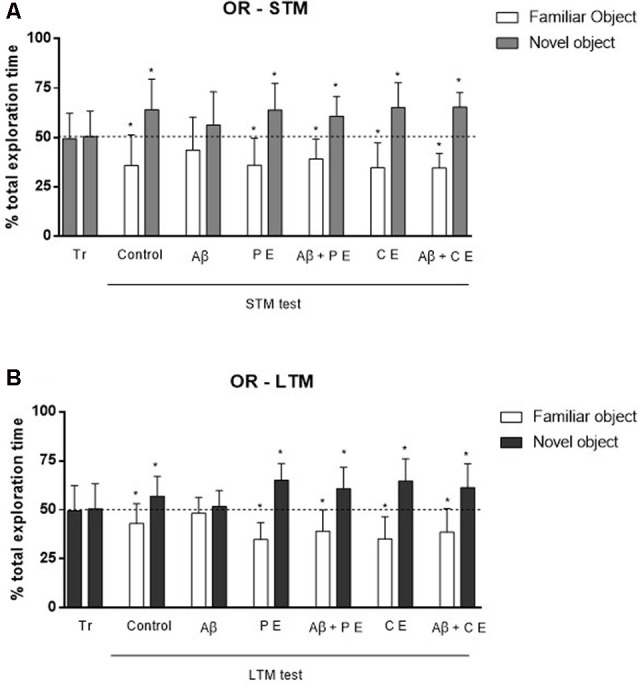
The hippocampal Aβ infusion promotes Object Recognition (OR) memory deficit. PEs and CEs can revert the OR memory deficit caused by hippocampal Aβ infusion. **(A)** OR short-term memory (STM). **(B)** OR long-term memory (LTM). *P < 0.05; one-sample Student’s *t*-test (theoretical mean 50%). Data are presented as mean ± SD (*n* = 11–12/group). Tr, training.

In the OR memory training session, the rats explored each object for a similar percentage of total exploration time (in the graphs, the data are presented as the mean of all groups: object *A* = 51.24 ± 10.75%, *B* = 48.76 ± 10.75%; *P* > 0.05 for all groups; Control: *t*_(10)_ = 2.11, *P* = 0.06; Aβ: *t*_(11)_ = 1.08, *P* = 0.30; PE: *t*_(11)_ = 0.70, *P* = 0.49; Aβ + PE: *t*_(10)_ = 1.61, *P* = 0.14; CE: *t*_(11)_ = 0.21, *P* = 0.83; Aβ + CE: *t*_(11)_ = 1.42, *P* = 0.18; Figures 2A,B, tr). This result was expected, since both objects were new to the animals.

In the STM test, the control animals spent more time exploring the novel object (Control: *t*_(10)_ = 0.30, *P* = 0.0127; Figure 2A), showing that they remembered familiar objects. The same was observed in the PE and CE groups, which spent more time exploring the novel object (PE: *t*_(11)_ = 3.56, *P* = 0.0045; CE: *t*_(11)_ = 4.18, *P* = 0.0015; Figure 2A). The Aβ infusion impaired STM, since the rats spent a similar percentage of time exploring both objects, familiar and novel (Aβ: *t*_(11)_ = 1.32, *P* = 0.21; Figure 2A). The 4 weeks of treatment reversed the deficits caused by Aβ neurotoxicity; the animals subjected to Aβ infusion and to PE or CE training were able to form OR STM, i.e., they explored the novel object more than 50% of the total exploration time (Aβ + PE: *t*_(10)_ = 3.59, *P* = 0.0049; Aβ + CE: *t*_(11)_ = 7.22, *P* = 0.001; Figure 2A).

Twenty-four hours after the training, rats in the Control, PE and CE groups explored the novel object for more than 50% of the total exploration time (Control: *t*_(10)_ = 2.28, *P* = 0.04; PE: *t*_(11)_ = 6.08, *P* = 0.0001; CE: *t*_(11)_ = 4.54, *P* = 0.0008; LTM; Figure 2B). The animals in the Aβ group presented impaired LTM since they spent approximately 50% of the total exploration time on each object (LTM; Aβ: *t*_(11)_ = 0.74, *P* = 0.47; Figure 2B). PE and CE were able to reverse the damage induced by the Aβ protein since the Aβ + PE and Aβ + CE groups spent more than 50% of the total exploration time exploring the novel object (Aβ + PE: *t*_(10)_ = 3.34, *P* = 0.0075; Aβ + CE: *t*_(11)_ = 3.28, *P* = 0.007; Figure 2B).

**Figure 3:**
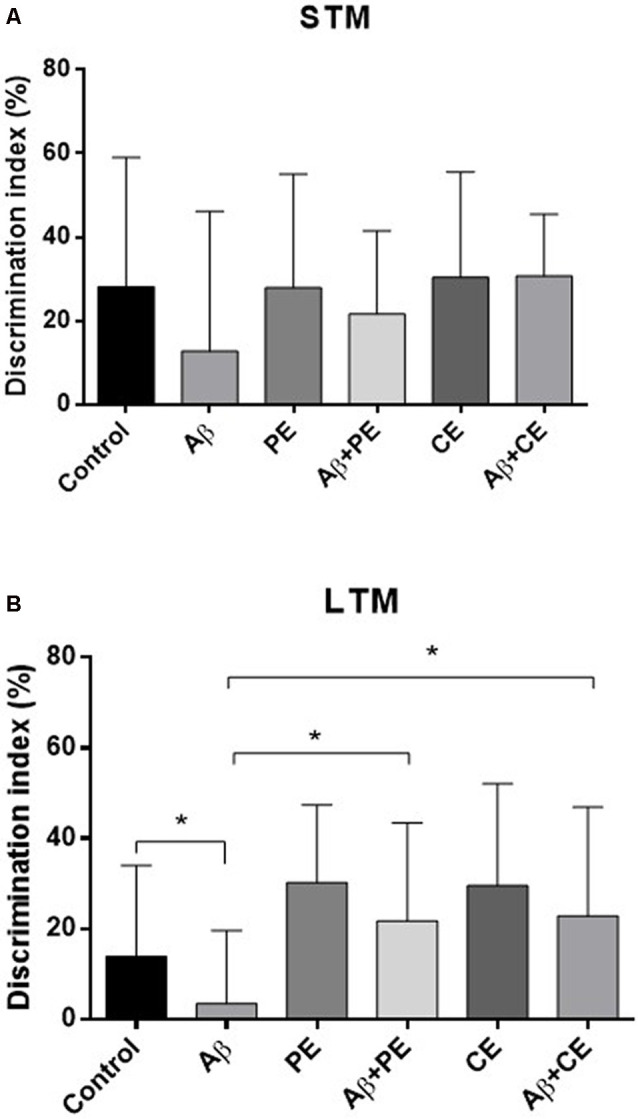
The PE and CE interventions overcome OR LTM, but not STM, memory discrimination index (DI) deficit induced by Aβ protein. *P < 0.05, Kruskal–Wallis followed by Mann–Whitney test. **(A)** STM DI. **(B)** LTM DI. Data are presented as mean ± SD (*n* = 11–12/group).

Considering the DI, used for comparison between the groups, no differences were found in STM test (*F*_(5,64)_ = 0.85; *P* = 0.51; Figure 3A). In contrast, significant differences were found in LTM test (*H*_(6)_ = 13.93; *P* = 0.016; Figure 3B). The animals of Aβ group presented lower DI compared to Control group (*U* = 30, *P* = 0.026). The interventions overcome memory deficits induced by Aβ protein (Aβ + PE vs. Aβ: *U* = 30, *P* = 0.026; Aβ + CE vs. Aβ: *U* = 32, *P* = 0.0204; Figure 3B). No differences were found between Control and PE group (*U* = 36; *P* = 0.06) and between Control and CE group (*U* = 40; *P* = 0.116).

#### Social Recognition (SR)

**Figure 4:**
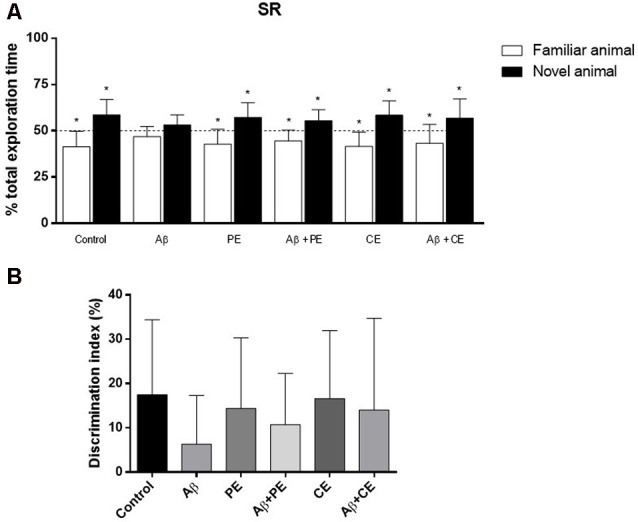
Effects of hippocampal Aβ infusion, physical and CEs on social recognition (SR) memory. **(A)** The hippocampal Aβ infusion promotes SR memory deficit. PE and CE can promote SR memory consolidation in rats that received intrahippocampal Aβ infusion. **(B)** There are no differences on SR DI between the groups. *P < 0.05; one-sample Student’s *t*-test (theoretical mean 50%). Data are presented as mean ± SD (*n* = 11–12/group).

In the SR memory test session, Control, PE and CE rats explored the novel rat for a longer percentage of time than the familiar rat (Control: *t*_(10)_ = 3.43, *P* = 0.0064; PE: *t*_(11)_ = 3.19, *P* = 0.0098; CE: *t*_(11)_ = 3.77, *P* = 0.0031; Figure 4A). Animals in the Aβ group, however, explored each rat for ~50% of the total exploration time (Aβ: *t*_(11)_ = 1.97, *P* = 0.07; Figure 4A). PE and CE reversed the deleterious effect of Aβ protein on SR memory, since the treated animals spent more than 50% of the total exploration time exploring the new rat (Aβ + PE: *t*_(10)_ = 3.11, *P* = 0.011; Aβ + CE: *t*_(11)_ = 2.29, *P* = 0.042; Figure 4A).

Considering the DI, used for comparison between the groups, no differences were found (*F*_(5,64)_ = 0.822; *P* = 0.538; Figure 4B).

### Biochemical Results

**Figure 5:**
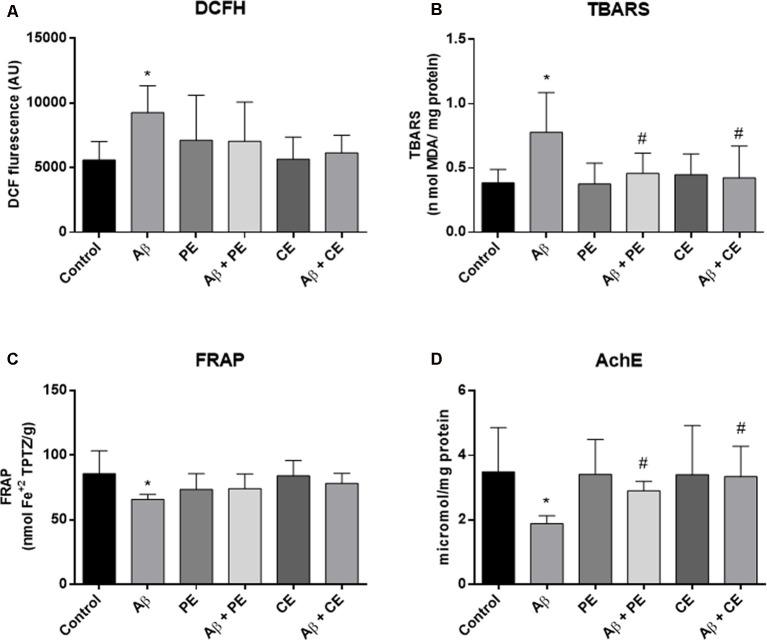
Aβ hippocampal infusion promotes reactive oxygen species (ROS; DCFH, **A**) and lipid peroxidation increase (TBARS, **B**); and promotes total antioxidant capacity **(C)** and acetylcholinesterase activity decrease **(D)**. The PE and the CE performed during 4 weeks reverted the lipid peroxidation **(B)**, and AchE activity **(D)** alterations. Data from ROS (DCFH, **A**) and TBARS **(B)** are presented as mean ± SD and were analyzed by ANOVA followed by Tukey’s test. Data from FRAP **(C)** and Acetylcholinesterase (AChE; **D**) are presented as median ± interquartile range and were analyzed by Kruskal–Wallis test followed by Dunn’s test. *P < 0.05, compared to control. ^#^*P* < 0.05, compared to Aβ.

We found differences between the groups in ROS levels, as measured by the DCFH test (*F*_(5,42)_ = 2.767, *P* = 0.03; Figure 5A). Aβ rats presented higher ROS levels than the control group (*P* = 0.033; Figure 5A). No significant differences in hippocampal ROS levels were observed among the other groups.

Differences among the groups were found (*F*_(5,42)_ = 4.464; *P* = 0.002; Figure 5B) in hippocampal lipid peroxidation (TBARS). The infusion of Aβ protein increased hippocampal lipid peroxidation in comparison to the control group (*P* = 0.004; Figure 5B). PE and CE reversed the lipid peroxidation increase induced by Aβ (*P* = 0.031 for Aβ vs. Aβ + PE; *P* = 0.012 for Aβ vs. Aβ + CE; Figure 5B).

Differences in total antioxidant capacity (i.e., ferric reducing/antioxidant power—FRAP) were observed among the groups (*H*_(6)_ = 16.44, *P* = 0.0057; Figure 5C). The infusion of Aβ resulted in lower total antioxidant capacity than that observed in the control group (*P* = 0.0144; Figure 5C). No significant differences were observed among the other groups.

The AChE activity was different between the groups (*H*_(6)_ = 18.70, *P* = 0.0022; Figure 5C). Aβ rats presented decreased acetylcholinesterase activity compared to the control group (*P* = 0.0080; Figure 5D). Aβ rats subjected to PE and CE presented higher AChE activity than Aβ rats not subjected to any intervention (*P* = 0.0321 for Aβ vs. Aβ + PE; *P* = 0.0092 for Aβ vs. Aβ + CE; Figure 5D).

### Histological Results

**Figure 6:**
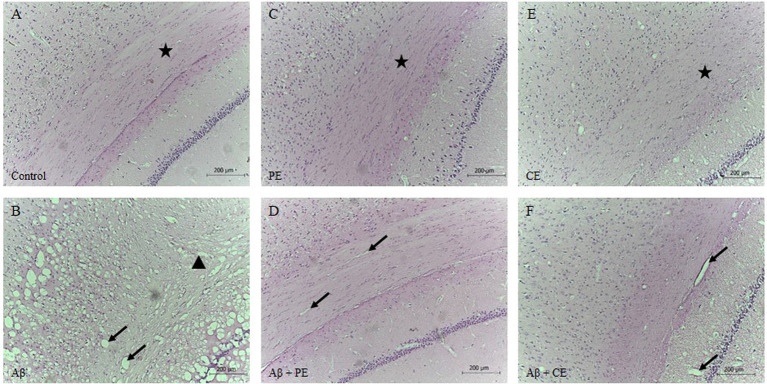
Control **(A)**, PE **(C)** and CE **(E)** groups presented standard hippocampal morphology with tissue organization. Infusion of Aβ promotes hippocampal disorganization, vacuoles formation, and neuronal tissue loss **(B)**. PE **(D)** and CE **(F)** improved hippocampal tissue morphology. Brain stained by hematoxylin-eosin (HE; magnification 10×). The arrows indicated vacuoles; the stars indicated tissue with normal organization, with cells in a parallel and layered position; and, the triangle indicated atrophy and tissue disorganization **(A)**: Control group; **(B)**: Aβ group; **(C)**: PE group; **(D)**: Aβ + PE group; **(E)**: CE group; **(F)**: Aβ + CE group.

Morphological differences in hippocampal tissue were observed among the groups. The control group presented a normal structure with the standard organization (Figure 6A). PE and CE training groups showed a normal structure, similar to the control group (Figures 6C,E). The infusion of the Aβ peptide promoted the formation of vacuole-like structures (indicated by arrows in Figure 6B), significant tissue disorganization, and clear neuronal tissue loss (indicated by triangles in Figure 6B). Aβ rats treated with physical and cognitive training showed improvements in hippocampal tissue disorganization due to Aβ infusion (Figures 6D,F).

## Discussion

The present results show that physical and CEs can promote recognition, short and LTM consolidation in animals with memory deficits induced by Aβ neurotoxicity. Also, physical and CEs were able to reverse long-term object recognition memory, decrease hippocampal lipid peroxidation, and restore hippocampal acetylcholinesterase activity altered by Aβ neurotoxicity. Still, PE and CE seem to promote a better morphological organization of the hippocampal tissue, which was altered after Aβ infusion.

It is difficult to study AD in humans since a precise diagnosis of this pathology is not simple or definitive. On the other hand, there are many available experimental models of AD, each one with benefits and limitations (Drummond and Wisniewski, 2017). An ideal model needs to mimic the lesions and symptoms of the disease in a way that is similar to the real situation (Duyckaerts et al., 2008). However, currently, no animal model reproduces all of the characteristics of AD. As the deposition of extracellular Aβ is one of the main AD features (Braak and Braak, 1988), brain infusion of Aβ protein is an important model that can contribute to the understanding of key aspects of AD biology since it mimics both the cognitive and the oxidative imbalance characteristics of AD.

It is important to consider that between the main regions where the Aβ deposits are in the AD patient brain are the hippocampus and cortex, structures that are associated with learning and memory function (Jing et al., 2009). Previous studies found increased hippocampal and cortical lipid peroxidation and protein oxidation in AD patients (Butterfield and Lauderback, 2002). In the same way, in this study, the hippocampal infusion of Aβ_25–35_ promoted hippocampal oxidative stress and damage (increased ROS levels and lipid peroxidation) and decreased the total antioxidant capacity and acetylcholinesterase activity in the hippocampus, leading to STM and LTM recognition deficits. Also, in a previous study, our group performed the histopathological analysis of the cerebral cortex of the rats submitted to the same Aβ hippocampal infusion model and we observed that the cerebral cortex of these rats presented intense deposition of amyloid plaques (Martinez-Oliveria et al., 2018).

The neuroprotective influence of PE on dementia is widely studied. Individuals who practice regular physical activity have a 30–40% reduced risk of AD development in comparison to physically inactive individuals (Aarsland et al., 2010; Williams et al., 2010). Several studies have demonstrated that aerobic PE, such as running or swimming exercise, improved memory in rats with Aβ-induced AD (Kim et al., 2014; Özbeyli et al., 2017; Prado Lima et al., 2018; Rossi Dare et al., 2019). Exercise-induced factors, including neurogenesis, synaptic plasticity, and increased cerebral blood flow, seem to promote beneficial effects on the brain (Tari et al., 2019). Following this literature, our results showed that PE was able to promote STM and LTM consolidation in Aβ rats, reversing oxidative stress disruption, and altering AChE activity.

As impressively as PE training, cognitive training also promoted short and long-term learning in Aβ rats. There is clinically significant evidence to support the effectiveness of CE (Cui et al., 2018), but little evidence about the possible mechanisms involved. In general, this type of intervention aims to maintain cognitive brain function as long as possible, reducing disabilities and improving patients’ quality of life (Zucchella et al., 2018). However, our results showed a direct effect of CE on cognition through its influence on hippocampal oxidative balance and AChE activity. A previous experimental study by our group showed that cognitive training was able to prevent the oxidative damage induced by the Aβ peptide in the same animal model (Rossi Dare et al., 2019); here, we demonstrated that CE can also have an important role after Aβ deposition.

It is important to highlight that both the physical and the cognitive training had a more prominent effect of LTM object recognition memory, since, for this type of memory, in addition to the ability to learn the task, it is noteworthy that the animals that received the Aβ infusion and underwent the different training had a significantly higher object DI than those that received beta-amyloid but did not perform any type of exercise. The same difference between the groups was not observed in STM OR. It is important to consider that STM and LTM consolidation involve different neurobiological mechanisms, as the increase of hippocampal protein and gene expression, that is observed in LTM consolidation but is not required for STM (Izquierdo et al., 1998). In this sense, the fact that these different types of memory involve some distinct neurobiological processes can justify the different effects observed.

Neurodegeneration related to AD is most pronounced in hippocampal cholinergic neurons, which are directly related to cognitive function (Gold, 2003; Kumar and Singh, 2015). In rat models of AD that present impairments in learning and memory, a loss of and damage to cholinergic neurons are observed (Klinkenberg and Blokland, 2010; Haider et al., 2015; Zhang et al., 2019). The degeneration of cholinergic neurons in AD promotes cholinergic hypofunction, which can result in a decrease in choline acetyltransferase and AChE activity in the hippocampus (Francis et al., 1999; Kaushal et al., 2019). Our data showed that Aβ promoted hippocampal tissue disorganization, vacuole formation, and neuronal loss, in addition to promoting a decrease in AChE activity. On the other hand, PE and CE were able to reverse the cholinergic hypofunction caused by Aβ infusion and to restore hippocampal tissue morphology.

Many studies address protection and prevention strategies to avoid AD. The most effective type of strategy seems to act before the pathology. However, we also need to think about treatment for patients who already have the disease, and currently, the alternatives are few. Since the symptoms of AD start to appear approximately 20 years after the onset of pathophysiological hallmarks (Dubois et al., 2014), when the patient discovers the disease, the biochemical changes are already advanced. Currently, pharmacological therapies available for AD contribute only to the temporary reduction of symptoms and slowing the progression of the disease (Habtemariam, 2019). Thus, strategies for treating the disease are important. Here, we present two interesting nonpharmacological options that are effective by acting on oxidative balance and cholinergic function.

In summary, the model of intrahippocampal Aβ infusion used in this study caused hippocampal oxidative stress and damage, decreased antioxidant capacity, altered hippocampal tissue morphology, and promoted short- and long-term deficits in recognition memory that mimic those described or suggested to occur in AD. Surprisingly, both PE and CE induced memory consolidation in animals that received Aβ infusion, which normally presents deficits. The effect of PE and CE is more prominent in long-term object recognition memory. Still, PE and CE reversed the lipid peroxidation and acetylcholinergic activity alterations induced by hippocampal Aβ. Therefore, both PE and CE have the potential to be included in the treatment of AD, since both interventions could be used in humans.

## Data Availability Statement

The datasets generated for this study are available on request to the corresponding author.

## Ethics Statement

The animal study was reviewed and approved by Animal Care and Use Committee from Federal University of Pampa—protocol n. 14/2017.

## Author Contributions

LD, AG, CS, LL, B-HN, and DD performed the experiments, analyzed the data, and wrote the manuscript. PM-C was responsible for the conceptualization of the study and supervision, analyzed the data, and wrote the manuscript.

## Conflict of Interest

The authors declare that the research was conducted in the absence of any commercial or financial relationships that could be construed as a potential conflict of interest.
